# *Helicobacter pylori* Infection in Cirrhotic Patients With Portal Hypertensive Gastropathy: A New Enigma?

**DOI:** 10.3389/fmed.2022.902255

**Published:** 2022-06-17

**Authors:** Sumaiah J. Alarfaj, Sally Abdallah Mostafa, Ramy A. Abdelsalam, Walaa A. Negm, Thanaa A. El-Masry, Ismail A. Hussein, Ahmed Mohamed El Nakib

**Affiliations:** ^1^Department of Pharmacy Practice, College of Pharmacy, Princess Nourah Bint Abdulrahman University, Riyadh, Saudi Arabia; ^2^Department of Medical Biochemistry and Molecular Biology, Faculty of Medicine, Mansoura University, Mansoura, Egypt; ^3^Department of Pathology, Faculty of Medicine, Mansoura University, Mansoura, Egypt; ^4^Department of Pharmacognosy, Faculty of Pharmacy, Tanta University, Tanta, Egypt; ^5^Department of Pharmacology and Toxicology, Faculty of Pharmacy, Tanta University, Tanta, Egypt; ^6^Department of Pharmacognosy and Medicinal Plants, Faculty of Pharmacy (Boys), Al-Azhar University, Cairo, Egypt; ^7^Department of Tropical Medicine, Faculty of Medicine, Mansoura University, Mansoura, Egypt

**Keywords:** gastric biopsy, histopathology, *Helicobacter pylori*, liver cirrhosis, PHG, prevalence

## Abstract

The relationship between *Helicobacter pylori* (*H. pylori*) infection and Portal hypertensive gastropathy (PHG) is still a debatable matter. The aim of this study is to find out how common *H. pylori* infection is in cirrhotic patients with PHG and to see if there’s a link between *H. pylori* infection and PHG severity. Out of 340 cirrhotic patients who had upper Gastrointestinal Tract (GIT) endoscopy for early varices screening, 160 cirrhotic patients were selected and divided into 2 groups; 80 cirrhotic patients with PHG (cases) and 80 cirrhotic patients without PHG (controls). Gastric biopsies were taken from all enrolled patients for histological evaluation for the presence or absence of *H. pylori* infection. *H. pylori* was found in 44 cirrhotic patients (55%) who had PHG (cases), compared to 22 cirrhotic patients (27.5%) who did not have PHG (controls). The prevalence of *H. pylori* infection was significantly higher in patients with PHG (*p* < 0.001). The severity of PHG was associated with *H. pylori* infection (*p* < 0.001). The response to eradication therapy of *H. pylori* infection was must better in patients without PHG (*p* = 0.045). By multi-variant analysis, *H. pylori* infection, splenic diameter, and portal vein diameter were independent predictors for PHG presence. After treating *H. pylori* infection in patients who tested positive for *H. pylori*, there was a significant reduction in PHG severity (*p* < 0.001). Patients with PHG have a greater prevalence of *H. pylori* infection. PHG is more severe in patients infected with *H. pylori*. To improve PHG severity, cirrhotic patients must have their *H. pylori* infection eradicated.

## Introduction

In patients with liver cirrhosis, gastrointestinal bleeding is a significant cause of hospitalization, morbidity, and mortality. It is caused by various etiologies, such as bleeding gastroesophageal varices, bleeding portal hypertensive gastropathy (PHG), portal hypertension-related intestinal and colonic lesions, and non-portal hypertension-related causes bleeding peptic ulcer, whether or not caused by *Helicobacter pylori* (*H. pylori*) infection (1, 2).

The prevalence of PHG in patients with portal hypertension has been observed to range from 20 to 80% (3, 4), with an incidence of acute upper GIT bleeding caused by PHG ranging from 2 to 12% (5).

Hematemesis, melena, and hematochezia are all symptoms of PHG, which can cause anemia due to either overt gastrointestinal bleeding or occult blood loss (6).

Mucosal edema, flat red spots, angiodysplasia-like lesions, pigmented black-brown spots, mucosal granularity, and reticulated mosaic-like pattern mucosa are all characteristics of PHG, which can be detected endoscopically (7, 8).

*H. pylori* is commonly seen in cirrhotic patients (9). However, it is significant in forming peptic ulcers in cirrhotic individuals (10). The impact of *H. pylori* on liver cirrhosis and PHG, on the other hand, is still up for debate (11, 12).

Portal hypertensive gastropathy appears to be caused by portal hypertension and changes in gastric microcirculation, which cause mucosal surface hypoxia and compromise epithelial cell integrity, most likely mediated by local factors such as nitric oxide overproduction, and oxygen-free radicals, endothelin-1, prostaglandins, and tumor necrosis factor α (13, 14).

However, other factors are associated with the existence and severity of PHG. Prior treatment for esophageal varices, the origin of portal hypertension (cirrhotic vs. non-cirrhotic), the severity of primary liver disease, and *H. pylori* infection are all things to think about (15).

*H. pylori* colonization of the stomach mucosa may indirectly affect PHG because colonization is related to inflammation, at least theoretically. *H. pylori* virulence factors increase mucosal inflammation by inducing the release of proinflammatory cytokines like tumor necrosis factor α (16).

This study aimed to determine how often *H. pylori* infection is in cirrhotic patients with PHG and to assess the possible association of *H. pylori* infection with PHG severity.

## Materials and Methods

### Study Design and Study Population

This transversal prospective case-control study was carried out in Tropical Medicine Department in collaboration with other departments at Mansoura University for over 1 year.

Out of 340 cirrhotic patients of different etiologies who had GIT endoscopy for early screening of varices, 160 patients were selected to be included in this study by the non-randomized method.

The patients were separated into 80 cirrhotic patients with PHG (cases) and 80 cirrhotic patients without PHG (control group). All participants in this study gave their written informed consent, and the Mansoura Faculty approved the study of Medicine’s ethical committee (Code number R.21.08.1416).

### Exclusion Criteria

**FIGURE 1:**
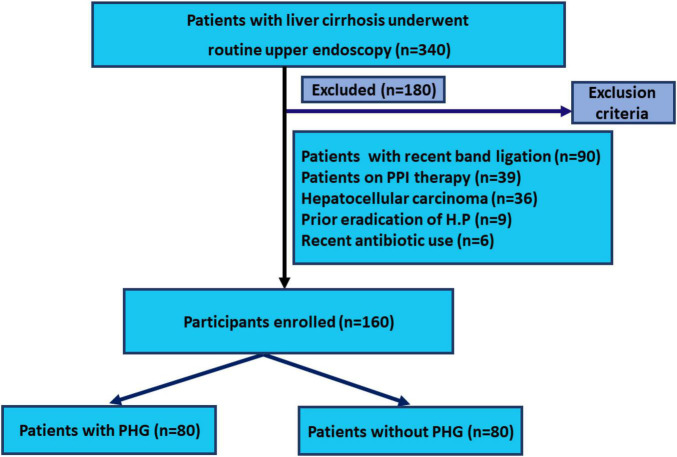
Consort-flow diagram of this study.

Patients with active peptic ulcer disease, patients with primary or secondary malignancy, patients who had previously undergone gastric surgery, had recently undergone injection sclerotherapy or band ligation for esophageal or gastric varices within 4 weeks before endoscopy, were on non-steroidal anti-inflammatory drugs or proton pump inhibitors, and had previously undergone *H. pylori* eradication therapy and had recently used antibiotics before endoscopy were all excluded. The study design of this case-control study and the steps that were performed are summarized in Figure 1 as a flowchart diagram.

### Diagnosis of Liver Cirrhosis and the Severity of Liver Disease

All patients and the control group were subjected to full history taking, clinical examination, laboratory investigations which included: Complete blood picture, serum creatinine, liver function tests (serum albumin, serum bilirubin, AST, ALT, and prothrombin time), virological markers (HBsAg, HCV Ab, and HIV) using ELISA test, Bilharzial Antibody test using ELISA test, Autoimmune markers (Anti-nuclear Antibody, Antismooth muscle Antibody, and Liver Kidney microsome type 1) and alpha-fetoprotein and radiological examination, which include Pelvi-Abdominal ultrasonography and dynamic computed tomography, if needed.

The diagnosis of liver cirrhosis was based on clinical examination, laboratory investigations, and radiological studies.

The severity of liver disease was measured using the Child-Pugh Classification and Model of End-stage Liver Disease (MELD) score.


MELD= 3.78⁢×⁢ln⁢[Bilirubin⁢(mg/dL)]+ 11.2⁢×⁢ln⁢[INR]+ 9.57⁢×⁢ln⁢[Creatinine⁢(mg/dL)]+ 6.43

The fibrosis severity was assessed using indirect markers of fibrosis such as Aspartate Aminotransferase (AST) to Platelet Ratio Index (APRI score).


$$\text{APRI}=\frac{\frac{\text{ASTLevel}}{\mathrm{AST}\,\mathrm{Upper}\,\mathrm{limit}\,\mathrm{of}\,\mathrm{Normal}}}{\mathrm{Platelet}\,\mathrm{Count}\,\left(10^{9}/L\right)}\,\text{×}\,100$$

And Fibrosis-4 (FIB-4 score).


$$\text{FIB4}=\frac{\mathrm{Age}\,\left(\text{Years}\right)\,\text{×AST}\,\left(U/L\right)}{\mathrm{Platelet}\,\mathrm{Count}\,\left(10^{9}/L\right)\,\text{×}\,\sqrt{\mathrm{ALT}\,\left(U/L\right)}}$$

### Upper Gastrointestinal Tract Endoscopy

After thorough preparation of the patient, the endoscopy was done using a disinfected upper gastrointestinal video scope Olympus EVIS 240 series Video-Endoscopy system for diagnosis of esophageal varices, gastric varices, diagnosis of portal hypertensive gastropathy, and its severity which was assessed using the PHG score method provided by Three-category system performed by Tanoue et al. (17) which classified PHG into: grade 1 (Mild): mild reddening congestive mucosa, grade 2 (Moderate): severe redness, and a fine reticular pattern separating areas of raised mucosa, and grade 3 (Severe): grade 2 plus point bleeding, and detecting signs suggesting *H. pylori* infection. Multiple antral gastric biopsies were taken by biopsy forceps.

### *Helicobacter pylori* Detection

**FIGURE 2:**
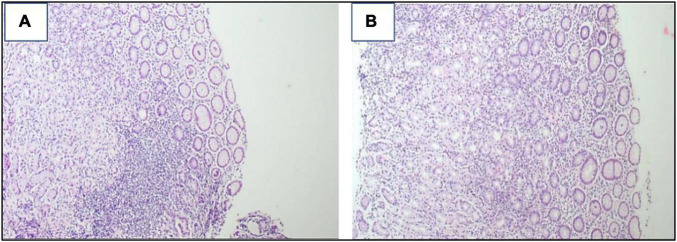
**(A,B)** Snips of gastric mucosa with widened lamina by mixed inflammatory cells, mainly lymphoplasmacytic with some neutrophils. Lymphoid aggregates are also seen ×200.

**FIGURE 3:**
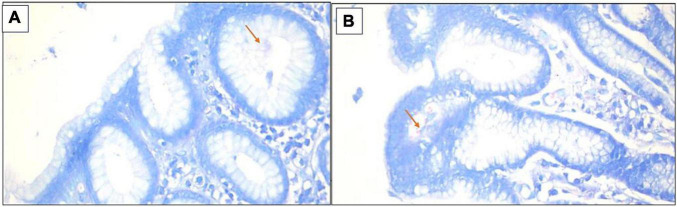
**(A,B)** Giemsa stain revealed the presence of *H. pylori* bacilli within foveolae ×400.

**FIGURE 4:**
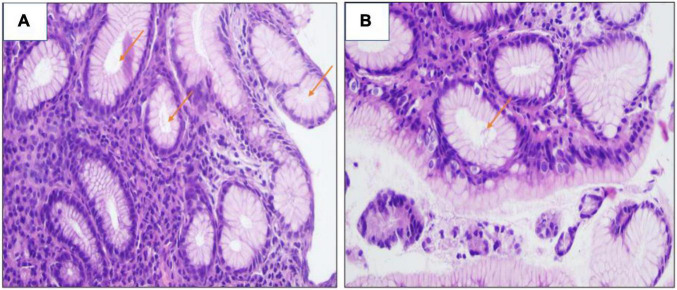
**(A,B)** H&E stain revealed the presence of *H. pylori* bacilli within foveolae at a mild density of ×400.

**FIGURE 5:**
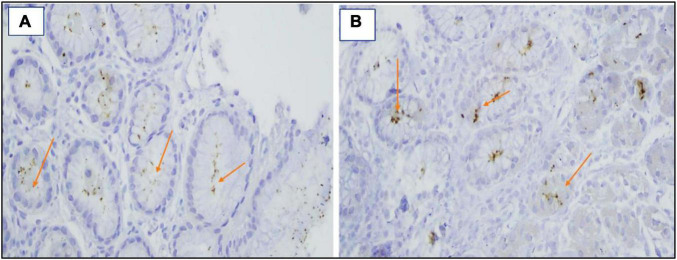
**(A,B)** Immunohistochemical staining revealed the presence of *H. pylori* bacilli with foveolae ×40.

For examination for the presence of *Helicobacter pylori* in this work, routinely processed, formalin-fixed, paraffin-embedded gastric antral tissues were cut into three-to-four-micron thick serial slices, mounted on grease-free slides, and submitted to Haematoxylin and Eosin (H&E) stain, Giemsa stain and then automated immunohistochemical staining was performed according to the manufacturer’s instructions using Dako Omnis auto-Stainer (Agilent, Denmark). Briefly, sections from paraffin-embedded Tissue Microarray (TMA) blocks were cut at 4 μm, deparaffinized with xylene, and rehydrated with graded alcohols. Heat is used for antigen retrieval using Envision Flex-target retrieval solution pH 9. Then tissue sections were incubated with antibodies against *H. pylori* (Antihuman mouse monoclonal, ready to use, code IR702, DAKO, Denmark) at 1:100 dilution for 60 min. The sections were incubated with Envision Flex/HRP, ready to use, goat secondary antibody against mouse IgG, SM802, DAKO, Denmark) for 30 min at room temperature, then counterstained with hematoxylin and examined (Figures 2–5).

### *Helicobacter pylori* Eradication in Patients With Positive Infection

According to American College of Gastroenterology guidelines (18), eradication of *H. Pylori* was performed using Clarithromycin triple therapy. This therapy was composed of PPI, Clarithromycin, and amoxicillin. The dose of PPI was standard or double dose twice daily. Clarithromycin dose was 500 mg twice daily, while amoxicillin was 1 g twice daily, and the duration of therapy was 14 days.

### Follow Up of the Patients

**FIGURE 6:**
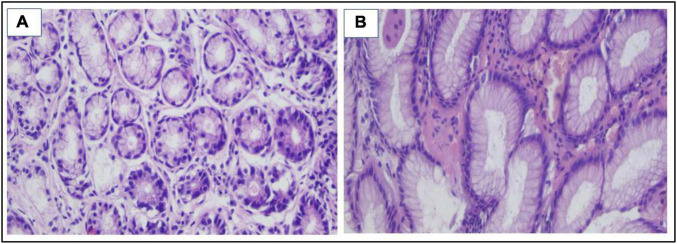
**(A,B)** H&E stain Snips of normal gastric mucosa with no detected *H. pylori* bacilli ×400 (evidence of eradication after triple therapy).

All the patients who were tested positive for *H. pylori* and given the triple therapy for elimination of *H. pylori* in both groups underwent follow-up upper GIT endoscopy 4 weeks after completion of treatment and after proton pump inhibitor therapy had been withheld for 1–2 weeks. The diagnosis of PHG and its severity was assessed using the PHG score method provided by the Three-category system (17) in patients with PHG. In addition, multiple antral gastric biopsies were taken using biopsy forceps to evaluate the response to therapy and confirm the eradication of *H. pylori* infection (Figure 6).

### Statistical Analysis

Microsoft Excel was used to enter and evaluate the data. After that, the data was imported into IBM/SPSS Inc.’s Statistical Package for the Social Sciences (SPSS 26.0, Chicago, IL) program for analysis. The study population’s baseline characteristics were reported as frequencies and percentages (%) in categorical data. In contrast, in the quantitative data, parametric data were presented as mean values, standard deviations (SD), and non-parametric data were presented as median and interquartile ranges.

The Chi-Square test (or Fisher’s exact test) was used to compare two or more independent groups of qualitative data for data comparison. An independent-Samples *t-*test was used to compare 2 parametric data sets for quantitative data.

Univariate and multivariate logistic regression analysis tested the dependent and independent risk factors for binary categorical variables. The *p-*values of less than 0.05 were deemed significant.

## Results

### Demographic Data and Baseline Characteristics of the Patients

The study enrolled 160 patients with liver cirrhosis: 80 patients with liver cirrhosis with an endoscopic diagnosis of PHG as cases and 80 patients with liver cirrhosis without PHG as a control group.

The mean age of cases was 47.98 ± 6.74 years, 68.8% were men, and 31.8% were women, while the mean age of the control group was 48.19 ± 7.82 years and 62.5% were men and 37.5% were women. There were no statistically significant differences between the cases and the controls as regards age, sex, etiology of liver cirrhosis, ascites, previous history of hepatic encephalopathy, child class, Platelet count, Aspartate Aminotransferase levels (AST), Alanine Aminotransferase levels (ALT), APRI score, and FIB-4 score.

There were statistically substantial differences between cases and the control group regarding MELD score, splenic diameter, and portal vein diameter (*p* = 0.015, < 0.001, and < 0.001, respectively) (Table 1).

**TABLE 1:** Baseline characteristics of cases and controls.

		Cases (PHG) (*n* = 80)	Control (No. PHG) (*n* = 80)	*p*
Age (years)		47.98 ± 6.74	48.19 ± 7.82	0.854
Gender	**Male**	55 (68.8%)	50 (62.5%)	0.405
	**Female**	25 (31.2%)	30 (37.5%)	
Etiology	**HCV**	76 (95%)	74 (92.5%)	0.808
	**HBV**	2 (2.5%)	3 (3.8%)	
	**AIH**	2 (2.5%)	3 (3.8%)	
Ascites		55 (68.8%)	55 (68.8%)	1
Previous history of encephalopathy		15 (18.8%)	16 (20%)	0.841
Child Pugh class A		15 (18.8%)	17 (21.2%)	0.806
B		30 (37.5%)	32 (40%)	
C		35 (43.8%)	31 (38.8%)	
Spleen diameter		15.98 ± 2.09	12.54 ± 0.87	<0.001*
Portal vein diameter		1.48 ± 0.23	1.37 ± 0.12	<0.001*
Platelets (*103)		150 (98–400)	150 (98–350)	0.934
AST (IU/L)		46.8 ± 4.23	46.80 ± 2.73	0.982
ALT (IU/L)		46.24 ± 5.73	46.69 ± 6.14	0.632
APRI		0.77 (0.28–1.3)	0.74 (0.31–1.61)	0.965
FIB-4		2.24 (0.68–4.74)	1.97 (0.83–4.18)	0.824
MELD score		17.68 ± 3.43	16.58 ± 2.09	0.015*

Quantitative data are expressed as mean and standard deviation and Median and IQR. Qualitative data are expressed as number (percent within the group). *p value is significant.

### Esophageal Varices Grades in Both Cases and Control Group

**FIGURE 7:**
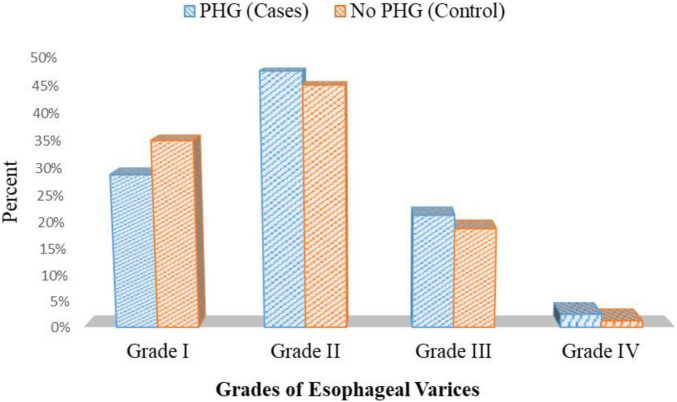
Esophageal varices grades in both cases and the control group.

Endoscopic examination of patients with PHG showed that 23 patients had grade I esophageal varices, 38 patients had grade II esophageal varices, 17 patients had grade III esophageal varices, and only two patients had grade IV esophageal varices. On the other hand, 28 patients had grade I esophageal varices, 36 patients had grade II esophageal varices, 15 patients had grade III esophageal varices, and only one patient had grade IV esophageal varices in patients without PHG *p* = 0.801 (Figure 7 and Supplementary Table 1).

### Presence of *Helicobacter pylori* Infection in Cases and Control Group

**FIGURE 8:**
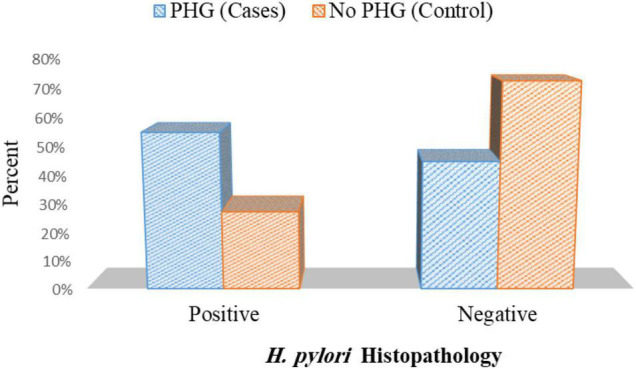
Presence of *H. pylori* histopathology in cases and controls.

As regards the existence of *H. pylori* in histopathology in cases and control group, *H. pylori* infection was observed in 44 patients out of 80 patients in cases (55%) and was detected only in 22 patients out of 80 patients in control (27.5%) *p*< 0.001 (Figure 8 and Supplementary Table 2).

### Association Between *Helicobacter pylori* Infection and Degree of Portal Hypertensive Gastropathy

**FIGURE 9:**
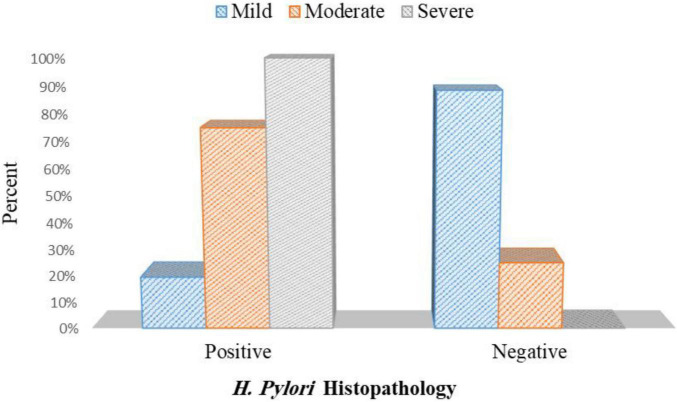
Association between the *H. pylori* histopathology and grade of PHG.

In patients with PHG, 8 out of 41 patients with mild PHG were positive for *H. pylori* infection (19.5%). Out of 12 patients with moderate PHG, 9 patients were positive for *H. pylori* infection (75%), and 27 patients out of 27 patients with severe PHG were positive for *H. pylori* infection (100%) *p*< 0.001 (Figure 9 and Supplementary Table 3).

### Response to Triple Therapy for Eradication of *Helicobacter pylori* in Both Cases and Control

**FIGURE 10:**
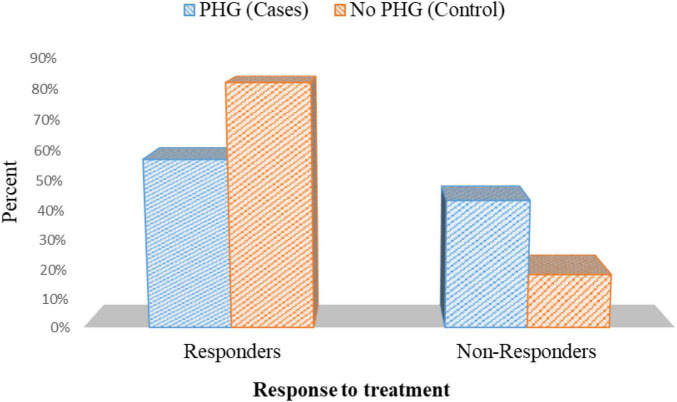
Response to treatment in cases with positive *H. pylori* histopathology in cases and controls.

In patients with PHG, 25 patients out of 44 patients who tested positive for *H. pylori* (56.8%) had responded to triple therapy. On the contrary, 18 out of 22 who tested positive for *H. pylori* (81.8%) responded to triple therapy in patients without PHG *p* = 0.045 (Figure 10 and Supplementary Table 4).

### Severity of Portal Hypertensive Gastropathy Before and After Treatment in the Cases Group

**FIGURE 11:**
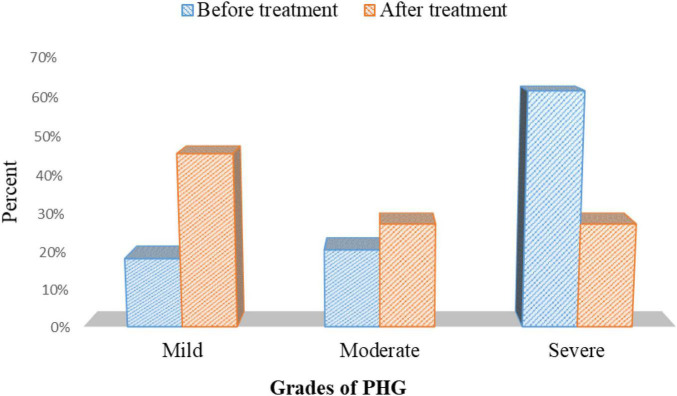
The severity of PHG before and after treatment in the cases group.

There was a statistically substantial improvement in PHG severity in patients with PHG after eradicating *H. pylori* using triple therapy *p* = 0.001 (Figure 11 and Supplementary Table 5).

### Univariate and Multivariate Analysis for Prediction of Portal Hypertensive Gastropathy Presence

By multi-variant analysis, *H. Pylori* infection, splenic diameter, and portal vein diameter were independent predictors for PHG presence. Odds Ratio = 3.22, 6.3, 2.4 and *p*-value = 0.001, < 0.001, and = 0.001, respectively (Table 2).

**TABLE 2:** Univariate and multi-variant analysis for prediction of PHG presence (dependent variable is PHG).

Variables	Univariate analysis	Multivariate analysis		
		*OR*	95% CI for OR	*p*-value
Age	0.853			
Male gender	0.406			
Ascites	1			
Hepatic encephalopathy	0.841			
*H. pylori* infection	0.001*	3.22	1.67–6.25	0.001*
Platelets	0.674			
AST	0.982			
ALT	0.630			
APRI score	0.938			
FIB4 score	0.771			
AIH	1 (Ref)			
HCV	0.810			
HBV	0.641			
Child A	1 (Ref)			
Child B	0.568			
Child C	0.600			
MELD score	0.017*	0.986	0.764–1.273	0.915
Spleen diameter	<0.001*	6.358	3.062–8.140	<0.001*
Portal vein diameter	<0.001*	2.415	1.644–3.760	0.001*

*p value is significant.

## Discussion

In individuals with portal hypertension, PHG isn’t the most prevalent cause of substantial upper GIT hemorrhage, but hemorrhage is the most severe consequence. PHG causes acute upper gastrointestinal tract bleeding in a wide range (2–12%) (5).

According to reports, 10% of PHGs produce anemia due to chronic blood loss, with more than 2% of patients experiencing severe bleeding (19).

The impact of *H. pylori* on liver cirrhosis and PHG, on the other hand, is still up for debate (11, 12). Our investigation aimed to determine the prevalence of *H. pylori* infection in cirrhotic patients with PHG and assess the possible association of *H. pylori* infection with PHG severity.

There was no statistically significant difference in baseline characters and etiology of liver disease between cases and controls in this investigation.

In terms of Child class, 15 patients (18.8%) were Child A cirrhosis, 30 patients (37.5%) were Child B cirrhosis, and 35 patients (43.8%) were Child C cirrhosis in patients with PHG. In comparison, 17 patients (21.2%) were Child A cirrhosis, 32 patients (40%) were Child B cirrhosis, and 31 (38.8%) patients were Child C cirrhosis in patients without PHG, and there was no substantial difference (*p* = 0.806). This is in line with the findings of Safwat et al. (20), Abbas et al. (21),and Eid et al. (12) (*p* = 0.59, 0.735, and 0.423, respectively). On the contrary, Kim et al. (22) and El-Toukhy et al. (23) found a significant link between PHG and Child score. The vulnerability of the gastric mucosa to harm caused by irritants such as ethyl alcohol, bile salts, aspirin, or classic non-steroid anti-inflammatory medications, and spontaneous bleeding may be influenced by liver dysfunction.

The MELD score was 17.68 ± 3.43 in patients with PHG and 16.58 ± 2.09 in patients without PHG (*p* = 0.015). This is consistent with two studies by El-Toukhy et al. (23) and El-Masry et al. (24), which found that the prevalence of *H. pylori* in HCV-infected patients rose considerably as the MELD score increased. This is in contrast to Safwat et al. (20), Abbas et al. (21), and Eid et al. (12), who found that there was no significant link between PHG and MELD scores (*p* = 0.468, 0.921, and 0.396, respectively). This could be explained by the limitations of the MELD scoring system.

The current investigation found that the spleen size was 15.98 ± 2.09 cm in patients with PHG and 12.54 ± 0.87 cm in patients without PHG (*p* < 0.001), and it was consistent with Kim et al. (22), who found that the mean spleen size was larger in cirrhotic patients with PHG. It differed, however, with Nashaat et al. (25), who found no statistically significant relationship between PHG and splenic diameter.

Regarding portal vein diameter, It was 1.48 ± 0.23 cm in patients with PHG, while it was 1.37 ± 0.12 cm in patients without PHG (*p* < 0.001). It was significantly dilated in PHG patients compared to non-PHG patients, consistent with Zardi et al. (26), who stated that the Portal vein was more dilated in cirrhotic patients with PHG, reflecting an increase in portal venous pressure with subsequent formation of the gastropathy. These findings may influence the hypothesis that *H. pylori* infection may contribute significantly to the development of PHG. PHG was thought to be caused by portal hypertension and changes in stomach microcirculation, which result in mucosal surface hypoxia and compromised epithelial cell integrity, leading to increased *H. pylori* colonization.

In our current research, the frequency of *H. pylori* infection was 55% in patients with PHG, while it was only 27.5% in patients without PHG (*p*< 0.001). This is in line with Safwat et al. (20), El-Toukhy et al. (23), and Sathar et al. (27).

In contrast, Hammad et al. (28) found that *H. pylori* did not link with PHG and that the difference between the prevalence of *H. pylori* and PHG was statistically insignificant. Infection with *H. pylori* in patients with PHG (70%) and the control group (63.3%) (*p* = 0.523).

Furthermore, Eid et al. (12) reported that the frequency of *H. pylori* infection was higher in PHG patients than in those without (34 vs. 10%). This is due to the stomach mucosa changes in cirrhosis, which may give a suitable media for *H. pylori* colonization, especially when associated with gastric mucosa swelling and severe hemorrhagic congestion resulting in high inducible nitric oxide.

Hu et al. (29) found that the link between *H. pylori* and PHG occurs because the gastric mucosa in PHG has thinner mucus and a higher pH due to decreased acid secretion and prostaglandin, a stomach protector, which weakens the gastric barrier. Furthermore, PHG has a decreased resting stomach trans-mucosal potential difference, which results in a decline in intracellular pH among mucosal cells, reduced mucus, and weakened gastric mucosal barrier, predisposing to mucosal lesions and providing ideal medium for *H. pylori* infection.

In this current research, in terms of the link between PHG severity and *H. pylori* infection, *H. pylori* were revealed to be substantially more common in patients with serious PHG than in those with milder PHG. This was in line with Hammad et al. (28) findings, which found a link between *H. pylori* infection and PHG severity, as *H. pylori* infection increases the release of proinflammatory cytokines such as tumor necrosis factor, which produce mucosal inflammation and predispose to the severity of PHG.

In contrast with these findings, Eid et al. (12), Pan et al. (15), and Abbas et al. (21) found no link between *H. pylori* and the severity of PHG in patients with cirrhosis (*p* = 0.079, 0.332, and 0.749, respectively).

In this current study, multivariate analysis for the prediction of PHG presence, splenomegaly, portal vein diameter, and *H. pylori* infection were significant independent predictors for the presence of PHG. This came in agreement with Hammad et al. (28), who documented a strong relation between *H. pylori* infection and PHG. Moreover, the splenic size was correlated significantly with the presence of PHG in cirrhotic patients, which agreed with Elwakil et al. (30), who mentioned a complex relationship between PHG and the presence of esophageal varices.

In this current research, after eradication therapy of *H. pylori* using the standard Clarithromycin-based triple therapy, the percentage of response to the treatment in patients with PHG was 56.8%. On the other hand, it was 81.8% in patients without PHG (*p* = 0.045). It may be because PHG has a low resting gastric trans-mucosal possible difference. This leads to a reduction in mucosal cell intracellular pH, a decrease in mucus, and a weakening of the stomach mucosal barrier, predisposing to mucosal lesions and resulting in appropriate media for *H. pylori* infection.

This study showed that the severity of PHG had improved after the elimination of *H. pylori* infection (*p* = 0.001).

This may be explained by the fact that the removal of *H. pylori* decreases the release of proinflammatory cytokines as tumor necrosis factors which reduces the mucosal inflammation and predisposes to the improvement of PHG severity.

To the best of our knowledge, this is the first study to look into the influence of *H. pylori* on PHG in this developing field of research. These findings have significant implications for our understanding of how *H. pylori* are linked to PHG pathogenesis and that its eradication substantially impacts the improvement of PHG severity.

This study has significant limitations, such as the fact that it is a single-center study with a smaller number of patients with a short duration of follow-up. Therefore, to validate the findings of the current investigation, more extensive multicenter trials with a diverse study population and a long period of follow-up of the patients would be required.

## Conclusion

Ultimately, patients with PHG have a greater prevalence of H. pylori infection. PHG is more severe in patients infected with H. pylori. To improve PHG severity, cirrhotic patients must have their H. pylori infection eradicated.

## Data Availability Statement

The original contributions presented in this study are included in the article/Supplementary Material, further inquiries can be directed to the corresponding author/s.

## Ethics Statement

The studies involving human participants were reviewed and approved by the Mansoura Faculty of Medicine’s Ethical Committee (Code number R.21.08.1416). The patients/participants provided their written informed consent to participate in this study.

## Author Contributions

AE: conceptualization. IH: data curation. SJA: formal analysis and project administration. TE-M: funding acquisition and supervision. SA, RA, and AE: investigation, methodology, and writing—original draft. SJA and IH: resources. WN and IH: software. WN, AE, and SJA: writing—review and editing. All authors have read and agreed to the published version of the manuscript.

## Conflict of Interest

The authors declare that the research was conducted in the absence of any commercial or financial relationships that could be construed as a potential conflict of interest.

## Publisher’s Note

All claims expressed in this article are solely those of the authors and do not necessarily represent those of their affiliated organizations, or those of the publisher, the editors and the reviewers. Any product that may be evaluated in this article, or claim that may be made by its manufacturer, is not guaranteed or endorsed by the publisher.

## References

[1] Holland-Bill L Christiansen C Gammelager H Mortensen R Pedersen L Sørensen H. Chronic liver disease and 90-day mortality in 21 359 patients following peptic ulcer bleeding–a nationwide cohort study. *Aliment Pharmacol Ther.* (2015) 41:564–72. 10.1111/apt.13073 25588862

[2] Alotaibi B Mokhtar FA El-Masry TA Elekhnawy E Mostafa SA Abdelkader DH Antimicrobial activity of *Brassica rapa* L. Flowers extract on gastrointestinal tract infections and antiulcer potential against indomethacin-induced gastric ulcer in rats supported by metabolomics profiling. *J Inflamm Res.* (2021) 14:7411. 10.2147/JIR.S345780 35002276 PMC8721290

[3] Sarin SK Shahi HM Jain M Jain AK Issar SK Murthy NS. The natural history of portal hypertensive gastropathy: influence of variceal eradication. *Am J Gastroenterol.* (2000) 95:2888–93. 10.1111/j.1572-0241.2000.03200.x 11051364

[4] Primignani M Carpinelli L Preatoni P Battaglia G Carta A Prada A Natural history of portal hypertensive gastropathy in patients with liver cirrhosis. *Gastroenterology.* (2000) 119:181–7. 10889167 10.1053/gast.2000.8555

[5] Merli M Nicolini G Angeloni S Gentili F Attili AF Riggio O. The natural history of portal hypertensive gastropathy in patients with liver cirrhosis and mild portal hypertension. *Am Coll Gastroenterol.* (2004) 99:1959–65. 10.1111/j.1572-0241.2004.40246.x 15447756

[6] Thuluvath PJ Yoo HY. Portal hypertensive gastropathy. *Am J Gastroenterol.* (2002) 97:2973–8. 12492178 10.1111/j.1572-0241.2002.07094.x

[7] Papazian A Braillon A Dupas J Sevenet F Capron J. Portal hypertensive gastric mucosa: an endoscopic study. *Gut.* (1986) 27:1199–203. 3781334 10.1136/gut.27.10.1199 PMC1433869

[8] Calés P Zabotto B Meskens C Caucanas J-P Vinel J-P Desmorat H Gastroesophageal endoscopic features in cirrhosis: observer variability, interassociations, and relationship to hepatic dysfunction. *Gastroenterology.* (1990) 98:156–62. 2293575

[9] Dore M Mura D Deledda S Maragkoudakis E Pironti A Realdi G. Active peptic ulcer disease in patients with hepatitis C virus-related cirrhosis: the role of *Helicobacter pylori* infection and portal hypertensive gastropathy. *Can J Gastroenterol.* (2004) 18:521–4. 10.1155/2004/150674 15372116

[10] Vergara M Calvet X Roqué M. *Helicobacter pylori* is a risk factor for peptic ulcer disease in cirrhotic patients. A meta-analysis. *Eur J Gastroenterol Hepatol.* (2002) 14:717–22. 12169979 10.1097/00042737-200207000-00002

[11] Al Mofleh IA. Does *Helicobacter pylori* affect portal hypertensive gastropathy? *Saudi J Gastroenterol.* (2007) 13:95. 10.4103/1319-3767.32186 19858622

[12] Eid KA Shawky MA Hassan AM Mohammed AQ Mohammed MI. Prevalence of *Helicobacter pylori* infection in patients with portal hypertensive gastropathy owing to liver cirrhosis in upper Egypt. *Al-Azhar Assiut Med J.* (2016) 14:109.

[13] Fontana RJ Sanyal AJ Mehta S Doherty MC Neuschwander-Tetri BA Everson GT Portal hypertensive gastropathy in chronic hepatitis C patients with bridging fibrosis and compensated cirrhosis: results from the HALT-C trial. *Am Coll Gastroenterol.* (2006) 101:983–92. 10.1111/j.1572-0241.2006.00461.x 16573786

[14] Kawanaka H Tomikawa M Jones MK Szabo IL Pai R Baatar D Defective mitogen-activated protein kinase (ERK2) signaling in gastric mucosa of portal hypertensive rats: potential therapeutic implications. *Hepatology.* (2001) 34:990–9. 10.1053/jhep.2001.28507 11679970

[15] Pan W-D Xun R-Y Chen Y-M. Correlations of portal hypertensive gastropathy of hepatitis B cirrhosis with other factors. *Hepatobiliary Pancreat Dis Int.* (2002) 1:527–31. 14607680

[16] Patel MK Trombly MI Kurt-Jones EA. Innate immune responses to *Helicobacter pylori* infection: an overview. *Methods Mol Biol.* (2012) 921:205–7. 23015506 10.1007/978-1-62703-005-2_23

[17] Tanoue K Hashizume M Wada H Ohta M Kitano S Sugimachi K. Effects of endoscopic injection sclerotherapy on portal hypertensive gastropathy: a prospective study. *Gastroint Endosc.* (1992) 38:582–5. 10.1016/s0016-5107(92)70522-7 1397916

[18] Chey WD Leontiadis GI Howden CW Moss SF. ACG clinical guideline: treatment of *Helicobacter pylori* infection. *Am Coll Gastroenterol.* (2017) 112:212–39. 10.1038/ajg.2016.563 28071659

[19] Chung WJ. Management of portal hypertensive gastropathy and other bleeding. *Clin Mol Hepatol.* (2014) 20:1. 10.3350/cmh.2014.20.1.1 24757652 PMC3992324

[20] Safwat E Hussein HA Hakim SA. *Helicobacter pylori* in Egyptian patients with HCV-related liver cirrhosis and portal hypertensive gastropathy: prevalence and relation to disease severity. *Life Sci J.* (2015) 12:168–73.

[21] Abbas Z Yakoob J Usman MW Shakir T Hamid S Jafri W. Effect of *Helicobacter pylori* and its virulence factors on portal hypertensive gastropathy and interleukin (IL)-8, IL-10, and tumor necrosis factor-alpha levels. *Saudi J Gastroenterol.* (2014) 20:120. 10.4103/1319-3767.129477 24705150 PMC3987152

[22] Kim DJ Kim HY Kim SJ Hahn TH Jang MK Baik GH *Helicobacter pylori* infection and peptic ulcer disease in patients with liver cirrhosis. *Korean J Intern Med.* (2008) 23:16. 18363275 10.3904/kjim.2008.23.1.16 PMC2686959

[23] El-Toukhy N Omar El-Farouk L Youssef M. *Helicobacter pylori* infection is associated with portalı hypertensive gastropathy in patients with liver cirrhosisı. *Afro Egypt J Infect Endem Dis.* (2021) 11:61–8.

[24] El-Masry S El-Shahat M Badra G Aboel-Nour MF Lotfy M. *Helicobacter pylori* and hepatitis C virus coinfection in Egyptian patients. *J Glob Infect Dis.* (2010) 2:4. 10.4103/0974-777X.59244 20300411 PMC2840963

[25] Nashaat EH Abd-Elaziz H Sabry M Ibrahim A. Non-endoscopic predictors of esophageal varices and portal hypertensive gastropathy. *Nat Sci.* (2010) 8:43–50.

[26] Zardi EM Ghittoni G Margiotta D Viera FT Di Matteo F Rossi S. Portal hypertensive gastropathy in cirrhotics without varices: a case–control study. *Eur J Gastroenterol Hepatol.* (2015) 27:91–6. 10.1097/MEG.0000000000000234 25386762

[27] Sathar SA Kunnathuparambil SG Sreesh S Narayanan P Vinayakumar KR. *Helicobacter pylori* infection in patients with liver cirrhosis: prevalence and association with portal hypertensive gastropathy. *Ann Gastroenterol.* (2014) 27:48. 24714519 PMC3959527

[28] Hammad OM Abu-Seif MA Ashour M Hifnawy T. Correlation of portal hypertensive gastropathy with *Helicobacter pylori* infection, liver dysfunction, hypersplenism and oesophageal varices. *Med J Cairo Univ.* (2009) 77:597–601.

[29] Hu J-K Li X-M Gu B-H Zhang F Li Y-M Chen H. *Helicobacter pylori* and portal hypertensive gastropathy. *Hepatobiliary Pancreat Dis Int.* (2018) 17:578–80. 30414714 10.1016/j.hbpd.2018.10.007

[30] Elwakil R Al Breedy AM Gabal HHA. Effect of endoscopic variceal obliteration by band ligation on portal hypertensive gastro-duodenopathy: endoscopic and pathological study. *Hepatol Int.* (2016) 10:965–73. 10.1007/s12072-016-9711-z 26932843

