# Supplementary material for: Helicobacter pylori Infection in Cirrhotic Patients With Portal Hypertensive Gastropathy: A New Enigma?
Source: Front Med (Lausanne). 2022 Jun 17;9:902255. doi: 10.3389/fmed.2022.902255 (PMC9254718; doi:10.3389/fmed.2022.902255)
Supplement: Supplementary file 1 [file Data_Sheet_1.docx]

*Helicobacter Pylori* infection in cirrhotic patients with portal hypertensive gastropathy: A new enigma?

**Sumaiah J. Alarfaj ^1,^†, Sally Abdallah Mostafa ^2,^†, Ramy A. Abdelsalam ^3^, Walaa A. Negm ^4,^*, Thanaa A. El-Masry ^5^, Ismail A. Hussein** **^6^, Ahmed Mohamed El Nakib ^7,^***

^1^ Department of Pharmacy Practice, college of pharmacy, Princess Nourah bint Abdulrahman University. P.O.Box 84428, Riyadh 11671, Saudi Arabia

^2^ Department of Medical Biochemistry and Molecular Biology, Faculty of Medicine, Mansoura University, Mansoura, 35511, Egypt; sallyabdallah@mans.edu.eg

^3^ Pathology Department, Faculty of Medicine, Mansoura University, Mansoura, 35511, Egypt; Dr_ramy_666@mans.edu.eg

^4^ Department of Pharmacognosy, Faculty of Pharmacy, Tanta University, Tanta 31527, Egypt

^5^ Department of Pharmacology and Toxicology, Faculty of Pharmacy, Tanta University, Tanta 31111, Egypt; thanaa.elmasri@pharm.tanta.edu.eg

^6^ Department of Pharmacognosy and Medicinal Plants, Faculty of Pharmacy (Boys), Al‐Azhar University,

Cairo 11884, Egypt; ismaila.hussein@azhar.edu.eg

**^7^** Department of Tropical Medicine, Faculty of Medicine, Mansoura University, Mansoura, 35511, Egypt; el_naqueeb@mans.edu.eg

*** Correspondence:
Walaa A. Negm**
walaa.negm@pharm.tanta.edu.eg

Orcid: 0000-0003-0463-8047

**Table S1.** Esophageal varices grades in both cases and the control group

|  |  | **PHG (cases)** | **NO PHG (control)** | ***P*** |
| --- | --- | --- | --- | --- |
| **Grades of esophageal varices** | **I** | 23 (28.75%) | 28 (35%) | 0.801 |
|  | **II** | 38 (47.5%) | 36 (45%) |  |
|  | **III**  **IV** | 17 (21.25%)  2 (2.5%) | 15 (18.75%)  1 (1.25%) |  |

**Table S2.** Presence of *H. pylori* histopathology in cases and controls

|  | | **Cases (PHG)**  **(n= 80)** | **Control (No PHG)**  **(n= 80)** | ***P*** **value** |
| --- | --- | --- | --- | --- |
| ***H. Pylori* histopathology** | **Positive** | 44 (55%) | 22 (27.5%) | <0.001* |
|  | **Negative** | 36 (45%) | 58 (72.5%) |  |

Qualitative data are expressed as number (percent within the group)

* : P-value is significant when ˂ 0.05.

**Table S3.** Association between the *H. pylori* histopathology and grade of PHG

|  | | **Mild**  **(n= 41)** | **Moderate**  **(n= 12)** | **Severe**  **(n= 27)** | ***P*** **value** |
| --- | --- | --- | --- | --- | --- |
| ***H. Pylori* histopathology** | **Positive** | 8 (19.5%) | 9 (75%) | 27 (100%) | <0.001* |
|  | **Negative** | 33 (88.5%) | 3 (25%) | 0 (0%) |  |

**Table S4.** Response to treatment in patients with positive *H. pylori* infection in cases and controls

|  | **Cases (PHG)**  **(n= 44)** | **Control (No PHG)**  **(n= 22)** | ***P* value** |
| --- | --- | --- | --- |
| **Responded to treatment** | 25 (56.8%) | 18 (81.8%) | 0.045* |
| **No response** | 19 (43.2%) | 4 (18.2%) |  |

Qualitative data are expressed as number (percent within the group)

* : P-value is significant when ˂ 0.05.

**Table S5.** Severity of PHG before and after treatment in the cases group

| **PHG severity** | **Before treatment**  **(n=44)** | **After treatment**  **(n=44)** | ***P* value** |
| --- | --- | --- | --- |
| **Mild** | 8 (18.2%) | 20 (45.4%) | 0.001* |
| **Moderate** | 9 (20.5%) | 12 (27.3%) |  |
| **Severe** | 27 (61.4%) | 12 (27.3%) |  |
